# Prevalence and Associated Factors of Dynapenia, Pre-Sarcopenia, and Sarcopenia in Korean Adults: A Cross-Sectional Epidemiological Study

**DOI:** 10.3390/medicina61040575

**Published:** 2025-03-24

**Authors:** Do-Youn Lee

**Affiliations:** College of General Education, Kookmin University, Seoul 02707, Republic of Korea; triptoyoun@kookmin.ac.kr; Tel.: +82-02-910-5540

**Keywords:** dynapenia, sarcopenia, pre-sarcopenia, prevalence, associated factors

## Abstract

*Background and Objectives*: This study examined the prevalence and risk factors of dynapenia, pre-sarcopenia, and sarcopenia among Korean adults using nationally representative data. *Materials and Methods*: A cross-sectional analysis was conducted using the 2022–2023 Korea National Health and Nutrition Examination Survey (KNHANES). Participants aged 20 years and older with available muscle strength and body composition measurements were included. Handgrip strength and skeletal muscle mass (measured via bioelectrical impedance analysis) were used to classify participants based on the Asian Working Group for Sarcopenia (AWGS) 2019 criteria. Logistic regression analysis identified associated risk factors. *Results*: The prevalence of pre-sarcopenia, dynapenia, and sarcopenia was 9.6%, 3.4%, and 1.6%, respectively. Pre-sarcopenia was most common across all age groups, while sarcopenia was primarily observed in older adults (*p* < 0.001). Age was a key predictor for all three conditions (*p* < 0.001), with sex differences—men had a higher prevalence of pre-sarcopenia (*p* = 0.014), whereas women showed higher rates of dynapenia (*p* = 0.003) and sarcopenia (*p* = 0.008). Low physical activity (*p* < 0.001), high stress (*p* = 0.021), and smoking (*p* = 0.012) were also significant risk factors. *Conclusions*: These findings highlight the importance of early identification and targeted interventions to prevent muscle deterioration. Public health strategies focusing on lifestyle modifications and sex-specific approaches may help mitigate the burden of sarcopenia and its related health complications in aging populations.

## 1. Introduction

Sarcopenia is a geriatric syndrome characterized by the progressive loss of skeletal muscle mass, accompanied by decreased muscle strength and physical performance [1]. While aging naturally leads to gradual declines in muscle mass and strength, sarcopenia represents a pathological condition where these losses occur beyond the expected rate and result in functional impairments [2,3,4,5,6,7]. The natural aging process involves a gradual decline in muscle mass and strength, starting as early as the third decade of life, typically at a rate of 1–2% per year [3,7]. However, this decline does not necessarily lead to functional impairments or increased health risks. This age-related muscle deterioration leads to functional limitations such as impaired mobility and disability and is associated with numerous adverse health outcomes including cardiovascular disease, osteoporosis, metabolic syndrome, and even increased mortality risk [2,3,4]. Muscle strength in particular has been identified as a key predictor of functional decline: even modest reductions in strength can substantially limit physical performance, leading to higher risks of falls and a loss of independence [4,5].

Muscle strength appears to decline more rapidly with age than muscle mass, suggesting that factors beyond muscle atrophy contribute to weakness [6,7]. To distinguish the loss of strength from the loss of mass, the term dynapenia was coined to describe an age-related reduction in muscle strength and power not solely explained by diminished muscle size [6]. On the other end of the spectrum, the early stage of muscle deterioration where muscle mass is low, but strength and function are still preserved, is often termed pre-sarcopenia [8]. These concepts have informed the recent consensus definitions of sarcopenia. For example, the Asian Working Group for Sarcopenia (AWGS) 2019 emphasizes low muscle strength as the primary criterion for sarcopenia diagnosis [9]. While European criteria (EWGSOP2) exist, AWGS 2019 is more suitable for Asian populations, including Koreans, due to its region-specific cutoffs and emphasis on low muscle strength. Thus, we adopted AWGS 2019 in this study. In practice, sarcopenia is now defined as low muscle strength plus low muscle mass. At the same time, dynapenia refers to isolated low strength without low mass, and pre-sarcopenia refers to isolated low muscle mass without functional impairment [8,10]. This staging underscores that the decline in muscle quality (strength) may precede or exceed the loss of muscle quantity in older adults [6,7].

Multiple factors contribute to the development of sarcopenia. Advancing age is the predominant risk factor—after the third decade of life, skeletal muscle mass declines by approximately 1–2% per year, with an even faster decline (up to ~3% per year) after the age of 65 [11]. Consequently, the prevalence of sarcopenia increases markedly with age: roughly 5–13% of adults over 60 have sarcopenia, rising to over 30% in those above 80 years [11,12].

Sarcopenia can in turn precipitate a cascade of negative outcomes. It is a major cause of frailty-related falls and fractures in the elderly, limits the ability to perform daily activities, and impairs quality of life [13,14]. A recent meta-analysis confirmed that sarcopenia significantly increases the risk of falls and physical disability in older adults [14,15]. On a biological level, poor nutrition (inadequate protein intake), sedentary behavior, smoking, and excessive alcohol consumption have all been linked to greater muscle loss and dysfunction [16,17]. By contrast, regular exercise (especially resistance training) and a protein-rich diet are protective and are key strategies for preventing sarcopenia progression [16,17]. Indeed, exercise interventions in sarcopenic older adults can improve muscle strength and mass, mitigating the syndrome’s impact [18]. Recently, in Korea and elsewhere, there has been a growing recognition of sarcopenia as an important public health issue, spurring active research into its epidemiology and interventions [19,20]. For instance, one nationwide study reported the prevalence of sarcopenia in Korean older adults (≥65 years) to be approximately 9.7% in men and 11.8% in women [19]. The early diagnosis and management of sarcopenia are critical: timely intervention can prevent further muscle deterioration, whereas delayed treatment leads to aggravated health problems and increased healthcare costs. Identifying risk factors and vulnerable subpopulations is therefore essential to inform preventive strategies and reduce the burden of sarcopenia [20,21].

Despite this growing awareness, most existing studies in Korea and other countries have focused on sarcopenia as a binary condition (present vs. absent) in older adults, without differentiating those with isolated strength loss or isolated muscle mass loss [2,19,22]. Many investigations have examined individual risk factors or specific comorbidities in select populations. For example, studies have explored the association between sarcopenia and cardiovascular disease in Korean elders, the impact of physical inactivity on sarcopenia in older men, and the prevalence of sarcopenia in patients with type 2 diabetes or anemia [3,23,24]. However, comprehensive analyses incorporating a broad range of demographic, lifestyle, and health-related factors in the general adult population are limited [25]. Moreover, a few prior studies have simultaneously evaluated dynapenia and pre-sarcopenia alongside sarcopenia. As a result, it remains unclear to what extent the risk factor profiles overlap or differ among these three conditions. Given that dynapenia and pre-sarcopenia may represent earlier or intermediate stages in the progression toward sarcopenia [26], understanding their epidemiology could inform early intervention efforts.

This study aimed to address these gaps by using nationally representative data from the Korea National Health and Nutrition Examination Survey (KNHANES) to investigate the prevalence and correlation of dynapenia, pre-sarcopenia, and sarcopenia among Korean adults. By distinguishing muscle strength loss from muscle mass loss in a general population, our cross-sectional analysis seeks to provide nuanced insights that can guide targeted public health strategies for preserving muscle health in an aging society.

## 2. Materials and Methods

### 2.1. Study Design and Participants

This study is a cross-sectional analysis of data from the KNHANES, a nationwide surveillance program conducted by the Korea Disease Control and Prevention Agency. This study utilized data from a KNHANES cycle in which muscle strength and body composition were measured for adult participants. KNHANES employs a complex, stratified multistage probability sampling design to obtain a representative sample of the Korean population. All participants provided informed consent, and the survey was approved by the institutional review board of the KDCA.

**Figure 1:**
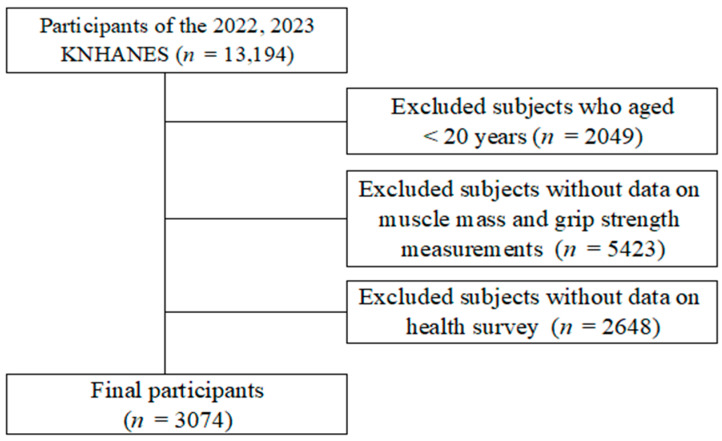
Participant selection process.

From the initial 2022–2023 KNHANES sample, participants were excluded based on the following criteria to ensure a well-defined study population. First, 2049 participants younger than 20 years were removed to focus on adults. Next, 5423 participants without muscle mass and grip strength measurements were excluded, as these variables are essential for identifying dynapenia, pre-sarcopenia, and sarcopenia. Additionally, 2648 participants lacking health survey data were removed, as demographic, lifestyle, and health-related information were necessary for analysis. After applying these criteria, a total of 3074 adults aged 20 to 80+ years were included in the final sample, with a nearly equal sex distribution. This selection process ensured a representative and comprehensive dataset for analyzing muscle impairment in Korean adults (Figure 1).

### 2.2. Definitions of Dynapenia, Pre-Sarcopenia, and Sarcopenia

The diagnostic criteria used in this study were based on the AWGS 2019 consensus recommendations. Muscle strength was assessed through handgrip strength, which was measured in kilograms (kg) using a standardized dynamometer operated by trained technicians. Participants performed the grip strength test with both hands, and the maximum value was used for analysis. According to the AWGS 2019 criteria, low muscle strength was defined as a handgrip strength of less than 28 kg for men or less than 18 kg for women. Skeletal muscle mass was measured using bioelectrical impedance analysis (BIA, Inbody 970, Biospace, Seoul, Republic of Korea), which was part of the KNHANES physical examination component. To assess muscle mass, appendicular skeletal muscle mass (ASM)—calculated as the sum of lean mass in both arms and legs—was normalized to height in meters squared (kg/m^2^) to derive the appendicular muscle mass index. Low muscle mass was defined as an ASM/height^2^ of less than 7.0 kg/m^2^ in men or less than 5.7 kg/m^2^ in women [9,27].

Based on these criteria, participants were classified into four mutually exclusive groups. The normal group included individuals whose muscle strength and muscle mass were both above the cutoff values, meaning they did not meet any criteria for sarcopenia. The dynapenia group consisted of individuals with low muscle strength but preserved muscle mass, indicating significant weakness despite maintaining muscle quantity. The pre-sarcopenia group included participants with low muscle mass but normal muscle strength, meaning they had a quantitative reduction in muscle mass but maintained normal strength and physical function. The sarcopenia group comprised individuals with both low muscle mass and low muscle strength, meeting the formal definition of sarcopenia, which is sometimes referred to in the literature as confirmed sarcopenia.

#### 2.2.1. Sociodemographic Factors

Sociodemographic factors were obtained using standardized self-reported questionnaires as part of the KNHANES and included sex, age, education level, marital status, and household income. Age was categorized into young (20–39 years), middle-aged (40–64 years), and older adults (65+ years). Education level was classified into four categories: elementary school or less, middle school, high school, and university (college) or higher. Marital status was dichotomized as living with a spouse (married or cohabiting) versus without a spouse (unmarried, divorced, widowed, or separated). Individual incomes were provided as quartiles (Q1 = lowest income quartile up to Q4 = highest quartile). This study used these quartile categories to represent socioeconomic status. Residential areas were categorized as urban vs. rural, based on the administrative district of residence [19,22].

#### 2.2.2. Health Status and Behavior Factors

Health-related factors were collected through standardized questionnaires and health examinations. Subjective health status was assessed by asking participants to rate their general health as good, moderate, or bad. Perceived stress level was measured on a 4-point scale in KNHANES and classified into “high” stress versus “low” stress. Smoking status was categorized as current smoker, former smoker, or never smoker. Alcohol intake was categorized as yes or no for current drinking based on the regular consumption of alcoholic beverages. Physical activity levels were assessed using the Global Physical Activity Questionnaire (GPAQ) guidelines from the World Health Organization (WHO). Total physical activity was calculated in MET-minutes per week (MET-min/week), considering activity intensity and duration across work, leisure, and travel domains. According to WHO standards, vigorous-intensity activities were assigned 8.0 METs, moderate-intensity activities, 4.0 METs, and travel-related activities, 4.0 METs. Participants were classified into low physical activity (LPA) and moderate-to-vigorous physical activity (MVPA) groups based on total weekly MET-min. The LPA group included those with less than 600 MET-min/week or no physical activity, while the MVPA group comprised individuals engaging in moderate activity (600–3000 MET-min/week) or vigorous activity (>3000 MET-min/week). For the analysis, 600 MET-min/week was used as the cutoff to differentiate between LPA and MVPA, ensuring a comprehensive evaluation of physical activity levels [2,19,28].

#### 2.2.3. Comorbid Health Conditions

Comorbid health conditions that could be associated with muscle status were also considered. Hypertension, diabetes mellitus, dyslipidemia, and abdominal obesity were included as binary covariates. Hypertension was defined as systolic blood pressure ≥130 mmHg or diastolic ≥85 mmHg or the current use of antihypertensive medications. Diabetes was defined by fasting plasma glucose ≥126 mg/dL or the current use of anti-diabetic medication. Among blood lipid abnormalities, we examined hypertriglyceridemia (triglyceride level ≥150 mg/dL or use of lipid-lowering medication) and low HDL cholesterol (HDL < 40 mg/dL in men or <50 mg/dL in women or on medication for low HDL). Abdominal obesity was assessed by waist circumference and defined as a waist ≥90 cm in men or ≥85 cm in women [3,29].

### 2.3. Statistical Analysis

All statistical analyses were conducted using IBM SPSS Statistics version 28.0 (IBM Corp., Armonk, NY, USA). We applied the sampling weights and design variables provided by KNHANES to account for the complex survey design and to obtain nationally representative estimates. The descriptive characteristics of the study population were summarized by muscle status group. Continuous variables were presented as means with standard errors and categorical variables as unweighted counts and weighted percentages. Group comparisons were made using the Rao–Scott chi-square test. To examine factors associated with dynapenia, pre-sarcopenia, and sarcopenia, multivariable logistic regression analysis was performed, adjusting for potential confounders including age, sex, education level, marital status, household income, residential area, subjective health status, stress, smoking, alcohol consumption, physical activity levels, and comorbid conditions (hypertension, diabetes, hypertriglyceridemia, low HDL-C, and abdominal obesity). All covariates were entered into the model simultaneously to assess independent associations. The results of the logistic regressions are presented as odds ratios (ORs) with 95% confidence intervals (CIs). A two-tailed *p* < 0.05 was considered statistically significant for all analyses.

## 3. Results

### 3.1. Prevalence of Dynapenia, Pre-Sarcopenia, and Sarcopenia

**Figure 2:**
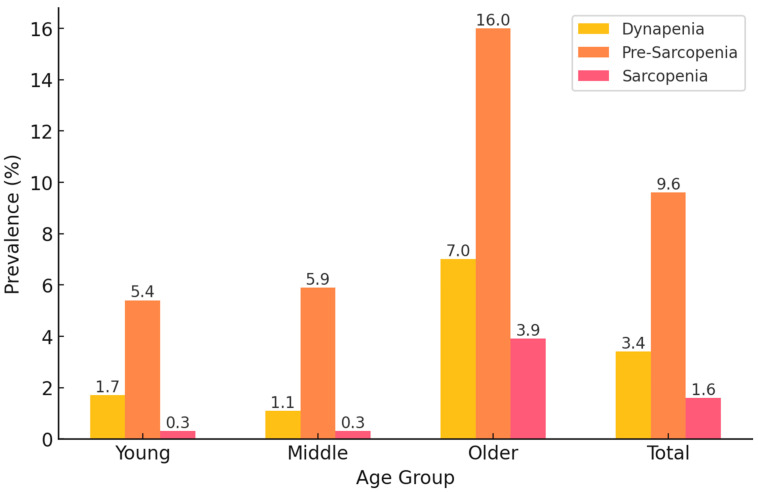
Prevalence of dynapenia, pre-sarcopenia, and sarcopenia in Korean adults.

Figure 2 shows the prevalence of dynapenia, pre-sarcopenia, and sarcopenia across different age groups (young, middle-aged, and older adults) in the study population. The results demonstrate a strong age-related trend, with the prevalence of all three conditions increasing with age.

Among young adults, pre-sarcopenia was the most common condition, affecting 5.4% of this group, followed by dynapenia (1.7%) and sarcopenia (0.3%). A similar pattern was observed in middle-aged adults, where pre-sarcopenia (5.9%) remained the most prevalent condition, while dynapenia (1.1%) and sarcopenia (0.3%) remained relatively rare. However, in older adults, the prevalence of all conditions increased markedly. Pre-sarcopenia was the most prevalent condition (16.0%), followed by dynapenia (7.0%) and sarcopenia (3.9%), indicating that muscle mass loss alone is more common than muscle strength loss or combined muscle impairment in this age group.

Overall, across all age groups, the total prevalence of pre-sarcopenia was 9.6%, dynapenia was 3.4%, and sarcopenia was 1.6% in the study population. These findings highlight that muscle impairment is strongly associated with aging, with pre-sarcopenia being the most frequent condition, particularly in older adults. This suggests that interventions targeting muscle mass preservation may be beneficial before significant declines in muscle strength occur.

### 3.2. Sociodemographic Characteristics of Participants

Table 1 presents the sociodemographic characteristics of participants by muscle condition. Age, sex, education level, and marital status differed significantly across groups (*p* < 0.001). Sarcopenia prevalence increased with age, with 88.3% of those with sarcopenia being older adults, compared to 31.7% in the normal group. Older adults also comprised 77.3% of the dynapenia and 62.0% of the pre-sarcopenia groups. Sex distribution varied significantly (*p* < 0.001); men were most prevalent in the pre-sarcopenia group (56.7%), while women dominated the dynapenia (72.2%) and sarcopenia (68.4%) groups. The education level was lowest in the sarcopenia and dynapenia groups, where 46.5% and 37.8% had only an elementary education, while 49.6% of the normal group had a university degree (*p* < 0.001). Marital status also showed significant differences (*p* < 0.001), with the highest proportion of partnered individuals in the normal group (85.9%) and the lowest in the sarcopenia group (62.5%). Income level (*p* = 0.801) and residential area (*p* = 0.256) did not differ significantly across groups.

### 3.3. Health Status and Health Behavior Characteristics of Participants

Table 2 presents the health status and health behavior characteristics of participants categorized as normal, dynapenia, pre-sarcopenia, and sarcopenia. Significant differences were observed in subjective health status, stress levels, smoking, alcohol consumption, physical activity, and certain comorbidities.

Subjective health status differed significantly across groups (*p* < 0.001), with good health being most reported in the normal group (37.0%) and least in the sarcopenia group (16.6%), while poor health was highest in the sarcopenia (33.6%) and pre-sarcopenia (25.3%) groups. Stress levels also varied (*p* = 0.037), being highest in the dynapenia (26.5%) and sarcopenia (24.1%) groups. Smoking status was significantly associated with muscle health (*p* < 0.001), with current smokers most common in the pre-sarcopenia (19.6%) group and least in the dynapenia (4.8%) and sarcopenia (5.3%) groups. Alcohol consumption also differed (*p* < 0.001), being highest in the normal group (54.7%) and lowest in the sarcopenia (20.0%) group. Physical activity levels were significantly different (*p* < 0.001), with LPA being most prevalent in the sarcopenia (97.9%), pre-sarcopenia (97.5%), and dynapenia (95.5%) groups. MVPA levels were extremely low, with only 2.1–4.5% engaging in moderate-to-vigorous physical activity. Among comorbidities, significant differences were observed in diabetes (*p* = 0.011), high triglycerides (*p* = 0.006), low HDL-C (*p* = 0.047), and abdominal obesity (*p* < 0.001). Diabetes was highest in the sarcopenia group (58.7%), while low HDL-C (29.8%) was also most frequent in this group. High triglycerides were more prevalent in the normal (26.8%) and dynapenia (23.9%) groups. Abdominal obesity was most common in dynapenia (53.7%), while hypertension showed no significant differences (*p* = 0.204).

### 3.4. Factors Associated with Dynapenia, Pre-Sarcopenia, and Sarcopenia

Table 3 presents the factors associated with dynapenia, pre-sarcopenia, and sarcopenia, expressed as odds ratios (ORs) with 95% confidence intervals (CIs). Several sociodemographic, health behavior, and comorbid conditions were significantly associated with muscle health status.

Age was a strong predictor of all three conditions, with older adults having significantly higher odds of dynapenia (OR = 5.426, *p* < 0.001), pre-sarcopenia (OR = 2.354, *p* < 0.001), and sarcopenia (OR = 7.695, *p* < 0.001). Men had increased odds of pre-sarcopenia (OR = 2.375, *p* < 0.001), but no significant association was found for dynapenia or sarcopenia. Lower education levels were associated with higher odds of pre-sarcopenia (*p* < 0.05), while marital status, income, and residential area showed no significant associations. Poor subjective health increased the odds of pre-sarcopenia (OR = 2.626, *p* = 0.001) and sarcopenia (OR = 3.412, *p* = 0.006). High stress was linked to dynapenia (OR = 2.084, *p* = 0.002), but not to the other conditions. Current smoking was associated with pre-sarcopenia (OR = 1.621, *p* = 0.039), while alcohol consumption was inversely related to pre-sarcopenia (OR = 0.670, *p* = 0.022) and sarcopenia (OR = 0.448, *p* = 0.015). Low physical activity significantly increased the odds of pre-sarcopenia (OR = 4.958, *p* = 0.001), while no associations were found for dynapenia or sarcopenia. Among comorbid conditions, abdominal obesity was a significant protective factor against pre-sarcopenia (OR = 0.107, *p* = 0.001) and sarcopenia (OR = 0.134, *p* = 0.001). High triglyceride levels were associated with lower odds of pre-sarcopenia (OR = 0.655, *p* = 0.040) but showed no significant association with dynapenia or sarcopenia. Other comorbidities, including hypertension, diabetes, and low HDL-C, were not significantly related to any of the three conditions.

## 4. Discussion

This study aimed to investigate the prevalence and associated factors of dynapenia, pre-sarcopenia, and sarcopenia in a nationally representative sample of Korean adults. The results indicate that muscle impairment is a prevalent issue, with distinct patterns across different subgroups. Approximately one in six Korean adults exhibited some form of muscle impairment: 4.2% had dynapenia, 10.7% had pre-sarcopenia, and 2.3% had sarcopenia. Advanced age was the strongest determinant of all three conditions, aligning with previous research on age-related skeletal muscle degeneration. Notably, distinct sex-based patterns were observed: dynapenia and sarcopenia were more prevalent in women, whereas pre-sarcopenia was more common in men. Additionally, modifiable lifestyle and psychosocial factors, including low physical activity, smoking, and high stress, were significantly associated with muscle impairment. Interestingly, abdominal obesity was inversely associated with pre-sarcopenia and sarcopenia, suggesting a complex interplay between body composition and muscle health.

The strong association between increasing age and all three conditions aligns with the natural progression of musculoskeletal aging. Previous studies have reported that skeletal muscle mass declines at a rate of 1–2% per year after the third decade of life, accelerating to nearly 3% per year after the age of 65 [11,30]. However, muscle strength declines more rapidly than muscle mass, which explains why dynapenia was prevalent in middle-aged and older adults even in the absence of sarcopenia [7]. This differential decline in muscle quantity and function suggests that neuromuscular factors, including motor unit remodeling, decreased neuromuscular activation, and reduced muscle fiber contractility, play a key role in age-related strength loss [31,32]. The higher prevalence of dynapenia compared to sarcopenia among older adults indicates that declines in muscle function may precede substantial muscle atrophy in some individuals [33,34]. These findings emphasize the importance of early interventions focusing on strength preservation, particularly through resistance training and neuromuscular activation strategies before substantial muscle mass loss occurs [35,36].

A notable finding of this study is the contrasting sex-related distribution across muscle impairment phenotypes. While men exhibited a significantly higher prevalence of pre-sarcopenia, women were more likely to develop dynapenia and sarcopenia. This pattern is consistent with previous research suggesting that men experience greater reductions in muscle mass with aging, whereas women exhibit earlier and more pronounced declines in muscle strength due to lower baseline reserves [37,38]. The higher prevalence of sarcopenia in women may be explained by postmenopausal hormonal changes, particularly the decline in estrogen, which has protective effects on muscle metabolism [39,40,41]. Additionally, as women generally have lower absolute muscle mass than men, they may be more vulnerable to sarcopenia once age-related muscle loss begins [42]. Conversely, men’s higher risk of pre-sarcopenia may reflect a tendency for muscle mass reduction without concurrent strength loss, possibly due to preserved muscle quality or delayed neuromuscular dysfunction [19,38,41]. These findings suggest that sex-specific interventions should be considered, with men at risk of pre-sarcopenia benefiting from muscle mass-preserving strategies (e.g., high-protein diets and resistance training), whereas women may require earlier strength-based interventions to prevent dynapenia and functional decline.

Beyond biological determinants, this study highlights the role of socioeconomic and behavioral factors in muscle impairment. Lower educational attainment was independently associated with pre-sarcopenia, which may reflect disparities in health literacy and access to preventive healthcare. Individuals with lower education levels may also be less aware of the importance of nutrition and physical activity in maintaining muscle function. Psychosocial stress showed a significant association with dynapenia, suggesting that chronic stress-related pathways, such as increased cortisol secretion and inflammatory responses, may contribute to accelerated strength loss [43]. Smoking was particularly associated with pre-sarcopenia, likely due to its detrimental effects on muscle protein synthesis, oxidative stress, and mitochondrial dysfunction [44]. In contrast, moderate alcohol consumption was inversely associated with sarcopenia, though this relationship requires careful interpretation given the well-documented negative effects of excessive alcohol intake on muscle function [45]. The strong association between low physical activity and all three conditions reinforces the importance of exercise in maintaining muscle health [46]. Our findings suggest that individuals with sarcopenia engage in significantly lower levels of moderate-to-vigorous physical activity compared to those without muscle impairment. Considering that resistance training is one of the most effective strategies for preserving both muscle mass and neuromuscular function, promoting physical activity—particularly among older adults—remains a critical public health priority [46].

This study also explored the association between metabolic health and muscle status. An unexpected finding was that abdominal obesity appeared to be protective against pre-sarcopenia and sarcopenia. One possible explanation is the “obesity paradox”, where excess fat mass, particularly in older adults, may serve as an energy reserve that helps preserve muscle mass and mitigate energy deficits, thereby delaying sarcopenia progression [47]. This phenomenon has been reported in other studies suggesting that mild-to-moderate adiposity may provide mechanical loading that contributes to muscle preservation. However, this interpretation must be approached with caution. While excess fat may initially help preserve muscle mass, chronic abdominal obesity is strongly associated with systemic inflammation, insulin resistance, and metabolic dysregulation, which can accelerate muscle degradation over time [48]. Additionally, higher fat mass may mask early-stage sarcopenia, leading to an underestimation of its prevalence in some populations, especially in the absence of functional assessments such as gait speed or the chair stand test. The observed association between diabetes and sarcopenia supports previous findings suggesting that insulin resistance and chronic inflammation contribute to muscle atrophy [49]. Hyperglycemia-induced oxidative stress and mitochondrial dysfunction are believed to impair muscle regeneration, leading to a progressive loss of muscle mass and strength [50]. Given these metabolic influences, the role of abdominal obesity in sarcopenia risk likely depends on multiple factors, including obesity severity, duration, and the presence of metabolic comorbidities. Future studies should not only explore overall body composition factors, such as total fat percentage and visceral fat, but also incorporate functional performance measures to provide a more comprehensive assessment of sarcopenia risk. These findings highlight the need for targeted interventions that address both muscle preservation and metabolic health as part of sarcopenia prevention strategies.

Compared to previous studies, our analysis provides a more nuanced perspective on muscle impairment by distinguishing between dynapenia, pre-sarcopenia, and sarcopenia. Most prior research has focused on sarcopenia as a binary condition, whereas our findings highlight the importance of evaluating isolated strength and mass loss as distinct clinical entities. The use of a nationally representative dataset enhances the generalizability of our results to the broader Korean population. Furthermore, our study supports the concept of “possible sarcopenia”, recently introduced by AWGS, which emphasizes the early identification of individuals at risk for muscle impairment. This highlights the potential utility of targeted screening measures in both clinical and community settings.

Despite its strengths, this study has several limitations. First, as this study is cross-sectional, it cannot establish causal relationships between risk factors and sarcopenia. Longitudinal studies are needed to confirm these associations and explore the progression of muscle impairment over time. Second, physical activity levels were self-reported, which may introduce recall bias. Objective measures such as accelerometry would provide more reliable insights. Third, we were unable to assess severe sarcopenia due to the lack of physical performance tests (e.g., gait speed and chair rise test). Moreover, as higher fat mass may mask the early stages of sarcopenia, the absence of functional assessments could have led to an underestimation of sarcopenia prevalence in this study. Future research should incorporate these functional assessments to better characterize sarcopenia severity and its effects on daily functioning and quality of life.

This study provides a comprehensive epidemiological assessment of dynapenia, pre-sarcopenia, and sarcopenia in Korean adults, offering valuable insights into their distinct risk factor profiles. Our findings emphasize the importance of early intervention to prevent muscle function decline, particularly among high-risk groups such as older adults, women, and individuals with low physical activity. Moving forward, prospective cohort studies are needed to clarify the causal pathways linking lifestyle factors and metabolic health to muscle impairment. Additionally, interventional trials examining the efficacy of targeted exercise programs, dietary modifications, and stress management strategies in preventing sarcopenia are warranted. Given Korea’s rapidly aging population, implementing community-based muscle health promotion programs will be essential in mitigating the burden of age-related muscle impairment and its associated adverse health outcomes.

## 5. Conclusions

This study analyzed the prevalence and risk factors of muscle impairment in Korean adults, revealing that pre-sarcopenia (9.6%) was the most common condition, followed by dynapenia (3.4%) and sarcopenia (1.6%). Older age, sex differences, and modifiable lifestyle factors were significant determinants of these conditions. Given these findings, early detection and intervention strategies, including routine screening, physical activity promotion, and dietary guidance, are essential to mitigating muscle decline, particularly in high-risk populations. Clinicians should focus on personalized prevention approaches to preserve muscle health and reduce sarcopenia-related complications.

## Figures and Tables

**Table 1:** Sociodemographic characteristics of participants according to dynapenia, pre-sarcopenia, and sarcopenia.

Factors	Categories	Normal (*n* = 2527)	Dynapenia (*n* = 140)	Pre-Sarcopenia (*n* = 335)	Sarcopenia (*n* = 72)	*p*
		M or %	M or %	M or %	M or %	
Age	Young	15.1	7.1	7.9	2.9	<0.001
	Middle	53.2	15.5	30.1	8.8	
	Older	31.7	77.3	62.0	88.3	
Sex	Male	44.6	27.8	56.7	31.6	<0.001
	Female	53.6	72.2	43.3	68.4	
Education level	Elementary	11.1	37.8	23.1	46.5	<0.001
	Middle	9.9	13.0	16.6	17.9	
	High	29.4	21.8	31.9	19.9	
	University	49.6	27.4	28.8	15.7	
Marital status	With	85.9	68.5	82.5	62.5	<0.001
	Without	14.1	31.5	17.5	37.5	
Individual income	Q1 (Lowest)	19.5	23.3	23.9	25.1	0.801
	Q2	24.3	24.2	24.8	27.7	
	Q3	26.3	25.1	24.0	20.8	
	Q4 (Highest)	29.8	27.4	27.4	26.5	
Residential area	Urban	84.9	77.4	84.4	81.0	0.256
	Rural	15.1	22.6	15.6	19.0	

**Table 2:** Health status and health behavior characteristics of participants according to dynapenia, pre-sarcopenia, and sarcopenia.

Factors	Categories	Normal (*n* = 2527)	Dynapenia (*n* = 140)	Pre-Sarcopenia (*n* = 335)	Sarcopenia (*n* = 72)	*p*
		M or %	M or %	M or %	M or %	
Subjective health status	Good	37.0	26.1	28.0	16.6	<0.001
	Moderate	47.7	53.0	46.7	49.8	
	Bad	15.3	20.9	25.3	33.6	
Stress level	High	21.7	26.5	15.5	24.1	0.037
	Low	78.3	73.5	84.5	75.9	
Smoking status	Current	14.6	4.8	19.6	5.3	<0.001
	Past	26.8	20.5	27.7	18.3	
	Non	58.5	74.7	52.7	76.4	
Alcohol status	Yes	54.7	33.8	42.6	20.0	<0.001
	No	45.3	66.2	57.4	80.0	
Physical activity	LPA	88.8	95.5	97.5	97.9	<0.001
	MVPA	11.2	4.5	2.5	2.1	
Comorbidity conditions						
Hypertension		28.4	31.9	34.2	29.1	0.204
Diabetes		41.6	51.1	45.8	58.7	0.011
High triglyceride		26.8	23.9	17.1	23.2	0.006
Low HDL-C		22.1	26	16.2	29.8	0.047
Abdominal obesity		40.7	53.7	9.5	15.4	<0.001

**Table 3:** Factors associated with dynapenia, pre-sarcopenia, and sarcopenia.

Categories		Dynapenia		Pre-Sarcopenia		Sarcopenia	
		OR (95% CI)	*p*	OR (95% CI)	*p*	OR (95% CI)	*p*
Age	Young	1 (reference)		1 (reference)		1 (reference)	
	Middle	0.646 (0.202–2.068)	0.010	0.923 (0.510–1.670)	0.009	0.635 (0.115–3.490)	0.010
	Older	5.426 (1.509–19.518)	<0.001	2.354 (1.239–4.471)	<0.001	7.695 (1.634–36.233)	<0.001
Sex	Male	0.588 (0.313–1.102)	0.098	2.375 (1.582–3.565)	<0.001	1.52 (0.654–3.533)	0.331
	Female	1 (reference)		1 (reference)		1 (reference)	
Education level	Elementary	1.094 (0.54–2.217)	0.803	2.065 (1.264–3.375)	0.004	2.273 (0.838–6.165)	0.107
	Middle	0.598 (0.276–1.297)	0.192	1.997 (1.212–3.29)	0.007	1.424 (0.558–3.636)	0.460
	High	0.786 (0.429–1.439)	0.433	1.58 (1.045–2.391)	0.031	1.197 (0.455–3.152)	0.716
	University	1 (reference)		1 (reference)		1 (reference)	
Marital status	With	0.711 (0.442–2.219)	0.159	0.937 (0.648–1.354)	0.726	0.535 (0.286–1.001)	0.051
	Without	1 (reference)		1 (reference)		1 (reference)	
Individual income	Q1 (Lowest)	1.233 (0.685–2.219)	0.485	1.132 (0.741–1.729)	0.568	1.242 (0.599–2.579)	0.561
	Q2	1.241 (0.673–2.289)	0.490	1.031 (0.671–1.585)	0.892	1.424 (0.726–2.794)	0.303
	Q3	1.060 (0.600–1.872)	0.843	0.952 (0.609–1.488)	0.828	0.918 (0.423–1.991)	0.828
	Q4 (Highest)	1 (reference)		1 (reference)		1 (reference)	
Residential area	Urban	0.687 (0.42–1.123)	0.134	1.308 (0.952–1.796)	0.098	1.076 (0.495–2.343)	0.854
	Rural	1 (reference)		1 (reference)		1 (reference)	
Subjective health status	Good	1 (reference)		1 (reference)		1 (reference)	
	Moderate	1.349 (0.846–2.15)	0.209	1.409 (0.971–2.045)	0.072	2.079 (0.995–4.344)	0.052
	Bad	1.173 (0.653–2.108)	0.594	2.626 (1.682–4.102)	0.001	3.412 (1.434–8.121)	0.006
Stress level	High	2.084 (1.338–3.247)	0.002	0.799 (0.55–1.162)	0.239	1.828 (0.948–3.524)	0.072
	Low	1 (reference)		1 (reference)		1 (reference)	
Smoking status	Current	0.495 (0.204–1.198)	0.119	1.621 (1.026–2.562)	0.039	0.436 (0.135–1.406)	0.164
	Past	0.933 (0.503–1.732)	0.825	0.792 (0.538–1.166)	0.236	0.606 (0.261–1.409)	0.244
	Non	1 (reference)		1 (reference)		1 (reference)	
Alcohol status	Yes	0.903 (0.552–1.478)	0.684	0.67 (0.476–0.942)	0.022	0.448 (0.236–0.852)	0.015
	No	1 (reference)		1 (reference)		1 (reference)	
Physical activity	Low	1.413 (0.596–3.351)	0.432	4.958 (1.941–12.664)	0.001	3.029 (0.394–23.304)	0.287
	Moderate–Vigorous	1 (reference)		1 (reference)		1 (reference)	
Comorbidities conditions							
Hypertension		0.731 (0.491–1.088)	0.122	1.175 (0.847–1.63)	0.336	0.646 (0.373–1.121)	0.120
Diabetes		1.13 (0.798–1.6)	0.491	1.085 (0.817–1.44)	0.575	1.614 (0.929–2.805)	0.090
High triglyceride		1.093 (0.653–1.83)	0.735	0.655 (0.437–0.981)	0.040	0.993 (0.474–2.077)	0.983
Low HDL-C		0.854 (0.536–1.362)	0.505	0.785 (0.501–1.231)	0.291	1.126 (0.588–2.155)	0.722
Abdominal obesity		1.228 (0.817–1.846)	0.325	0.107 (0.073–0.157)	0.001	0.134 (0.065–0.28)	0.001

## Data Availability

All data were anonymized and can be downloaded from the website (https://knhanes.kdca.go.kr/knhanes, accessed on 5 February 2025).
